# In Vitro Antimicrobial Activities of Organic Acids and Their Derivatives on Several Species of Gram-Negative and Gram-Positive Bacteria

**DOI:** 10.3390/molecules24203770

**Published:** 2019-10-19

**Authors:** Lauren Kovanda, Wen Zhang, Xiaohong Wei, Jia Luo, Xixi Wu, Edward Robert Atwill, Stefan Vaessen, Xunde Li, Yanhong Liu

**Affiliations:** 1 Department of Animal Science, University of California, Davis, CA 95616, USA; llkovanda@ucdavis.edu; 2 School of Life Science, Ningxia University, Yinchuan 750021, China; nx_zhangwen@163.com (W.Z.); sophia920209@163.com (J.L.); wuxixivip@163.com (X.W.); 3 School of Veterinary Medicine, University of California, Davis, CA 95616, USA; xhcwei@ucdavis.edu (X.W.); ratwill@ucdavis.edu (E.R.A.); 4 Perstorp Waspik BV, 5165 NH Waspik, The Netherlands; Stefan.Vaessen@perstorp.com

**Keywords:** antimicrobial effects, Gram-negative bacteria, Gram-positive bacteria, minimum inhibitory concentration, organic acids

## Abstract

The objective of this study was to determine the in vitro antimicrobial activity of several organic acids and their derivatives against Gram-positive (G+) and Gram-negative (G−) bacteria. Butyric acid, valeric acid, monopropionin, monobutyrin, monovalerin, monolaurin, sodium formate, and ProPhorce—a mixture of sodium formate and formic acid (40:60 *w*/*v*)—were tested at 8 to 16 concentrations from 10 to 50,000 mg/L. The tested bacteria included G− bacteria (*Escherichia coli, Salmonella enterica* Typhimurium, and *Campylobacter jejuni*) and G+ bacteria (*Enterococcus faecalis, Clostridium perfringens, Streptococcus pneumoniae*, and *Streptococcus suis*). Antimicrobial activity was expressed as minimum inhibitory concentration (MIC) of tested compounds that prevented growth of tested bacteria in treated culture broth. The MICs of butyric acid, valeric acid, and ProPhorce varied among bacterial strains with the lowest MIC of 500–1000 mg/L on two strains of *Campylobacter*. Sodium formate at highest tested concentrations (20,000 mg/L) did not inhibit the growth of *Escherichia coli*, *Salmonella* Typhimurium, and *Enterococcus faecalis*, but sodium formate inhibited the growth of other tested bacteria with MIC values from 2000 to 18,800 mg/L. The MIC values of monovalerin, monolaurin, and monobutyrin ranged from 2500 to 15,000 mg/L in the majority of bacterial strains. Monopropionin did not inhibit the growth of all tested bacteria, with the exception that the MIC of monopropionin was 11,300 mg/L on *Clostridia perfringens*. Monolaurin strongly inhibited G+ bacteria, with the MIC value of 10 mg/L against *Streptococcus pneumoniae*. The MIC tests indicated that organic acids and their derivatives exhibit promising antimicrobial effects in vitro against G− and G+ bacteria that are resistant to antimicrobial drugs. The acid forms had stronger in vitro antimicrobial activities than ester forms, except that the medium chain fatty acid ester monolaurin exhibited strong inhibitory effects on G+ bacteria.

## 1. Introduction

Antimicrobial drugs or antibiotics were discovered about a century ago and have been used widely in human and animal medicine, as well as in animal production. Antimicrobial growth promoters are antibiotics administered at low and subtherapeutic doses, which can enhance disease resistance and growth of animals [1,2]. However, there are growing concerns for the development of antimicrobial resistance and the potential transmission of antibiotic resistance genes in bacteria from livestock production to human beings. In the United States, therefore, the application of antibiotics as growth promoters was completely banned in the livestock industry starting January 2017 [3]. The urgent need for developing nutritional strategies or exploring bioactive compounds that may partially or completely replace antibiotics as growth promoters for food-producing animals has remarkably increased in the livestock industry.

Organic acids and salts of acids have been widely used in animal feed as acidifiers to modify the intestinal environment as well as to enhance nutrient digestibility [4]. The most commonly used acids include formic acid, citric acid, benzoic acid, carboxylic acids, and salts of short chain fatty acids (SCFAs) [5,6]. Recently, combinations of organic acids and medium chain fatty acids (e.g., lauric acid) have also demonstrated synergistic benefits on animal intestinal health and performance, compared with the individual products [7]. In general, antimicrobial activity has been claimed or suggested as one of the primary mechanisms of action through which organic acids could enhance animal health [8,9,10]. It is theorized that organic acids in their un-dissociated and uncharged state are capable of bypassing bacterial cell membranes due to their lipophilic nature [11]. Upon entering the more alkaline interior of a bacterium, the anion and proton from organic acids may have deleterious effects on the bacterium by increasing osmotic stress and disrupting important biomolecule synthesis, which finally causes bacterial death [12,13,14].

Although significant health benefits have been identified on SCFAs in vitro, direct addition of them in animal feed is limited because of their pungent odor and unpalatable flavor [15,16]. Therefore, SCFAs have been further processed as salt forms in combination with calcium or sodium, or as esterified forms before addition to animal feed [17,18,19]. Naturally, these products are more stable and/or pleasant compared with SCFAs [20]. An additional advantage of esterified SCFAs is that they could escape gastric digestion before reaching the small intestine of animals [21]. Many other organic acid derivatives require further investigation because they may exhibit antimicrobial activities and therefore could be added to animal feed as alternatives to antibiotics. Thus, the objective of the current study was to determine in vitro antimicrobial activity of several organic acids and their derivatives against Gram-positive (G+) and Gram-negative (G−) bacteria that were specifically selected due to their importance in the livestock industry.

## 2. Results

### 2.1. Organic Acids and Their Derivatives

All organic acids and their derivatives are liquid at ambient temperature, except for sodium formate and monolaurin. The specific gravity of monopropionin, monobutyrin, monovalerin, butyric acid, valeric acid, and ProPhorce were 1.30, 1.03, 1.01, 0.94, 0.92, and 1.36 g/mL, respectively. Monovalerin, monobutyrin, monopropionin, and monolaurin are monoglycerides and are not water-soluble. The chemical structures for individual organic acids and their derivatives are listed in Table 1.

### 2.2. Organic Acids and Their Derivatives Against G− Bacteria

The minimum inhibitory concentration (MIC) values of butyric acid against G− bacteria were 2300 or 2500 mg/L for *Escherichia coli (E. coli*; ATCC 25922 and F18) and *Salmonella enterica* Typhimurium (*S.* Typhimurium; ATCC 14028 and ID# 4286) (Table 2). The lowest MIC values of butyric acid were observed in *Campylobacter jejuni* (*C. jejuni*) with 500 mg/L for Campy 8DLIS D12-1 and 800 mg/L for ATCC 33560, respectively. The MIC values of valeric acid and ProPhorce were approximately 2000 to 2800 mg/L against *E. coli* and *S.* Typhimurium strains, and 500 to 1000 mg/L against *C. jejuni* (ATCC 33560) and *C. jejuni* (Campy 8DLIS D12-1), respectively. Sodium formate at the highest tested concentrations (20,000 mg/L) did not inhibit the growth of *E. coli* or *S.* Typhimurium strains. However, sodium formate inhibited the growth of both *C. jejuni* strains with a MIC value of 2000 mg/L.

Monobutyrin did not inhibit *C. jejuni* (ATCC 33560) even at the highest concentration of 50,000 mg/L. The MIC values of monobutyrin against other G− bacteria were between 10,000 and 15,000 mg/L. Monopropionin at 10,000 mg/L inhibited the growth of G− bacteria tested in the assays. Monovalerin had MIC values of 6700 and 5000 mg/L against *E. coli* strains, 10,000 and 15,000 mg/L against *S.* Typhimurium strains, and 2500 and 3700 mg/L against *C. jejuni* strains. Monolaurin had a MIC value of 10,000 mg/L against all *E. coli* and *S.* Typhimurium strains, and 5000 mg/L against *C. jejuni* (Campy8DLIS D12-1). The lowest MIC value of monolaurin was 600 mg/L against *C. jejuni* (ATCC 33560).

Antimicrobial susceptibility to common antimicrobial drugs of tested G− bacterial strains are shown in Table 3. Both reference and wild strains of *E. coli* and *Salmonella* were resistant to multiple drugs (i.e., ≥3 drugs). With the limited available MIC interpretive criteria, the susceptibility of *Campylobacter* to tested drugs was mostly undermined. However, both strains were resistant to ciprofloxacin, gentamicin, and tetracycline.

### 2.3. Organic Acids and Their Derivatives Against G+ Bacteria

The MIC values of butyric acid and valeric acid were both 2000 mg/L for *Enterococcus faecalis* (*E. faecalis*), 1200 and 1300 mg/L for *Clostridium perfringens* (*C. perfringens*), 700 and 1000 mg/L for *Streptococcus pneumoniae* (*S. pneumoniae*), and 700 and 1000 mg/L for *Streptococcus suis* (*S. suis*; Table 4). The MIC values of sodium formate were 11,000 to 18,800 mg/L against *C. perfringens* and two *Streptococcus* strains, but 20,000 mg/L sodium formate did not inhibit the growth of *E. faecalis*. The MIC values of ProPhorce were 1000 mg/L against *E. faecalis*, *C. perfringens*, or *S. pneumoniae*, and 1900 mg/L against *S. suis*.

Monobutyrin and monovalerin had similar antimicrobial activities on G+ bacteria. The MIC values of monobutyrin and monovalerin were 10,000 and 10,000 mg/L for *E. faecalis*, 2600 and 3100 mg/L for *C. perfringens*, 7700 and 2400 mg/L for *S. pneumoniae*, and 7800 and 2000 mg/L for *S. suis*. Monopropionin at the highest tested concentrations (25,000 mg/L) did not inhibit the growth of two *Streptococcus* strains. However, monopropionin inhibited the growth of *E. faecalis* and *C. perfringens* with MIC values of 10,000 and 11,300 mg/L, respectively. The MIC values of monolaurin were 500 mg/L for *E. faecalis*, 300 mg/L for *C. perfringens*, 10 mg/L for *S. pneumoniae*, and 400 mg/L for *S. suis*.

Susceptibility to commonly used antimicrobial drugs of tested G+ bacteria strains are shown in Table 5. *E. faecalis* (ATCC 29212) exhibited broad resistance to most tested antimicrobial drugs in this trial. With the available MIC interpretive criteria, *C. perfringens* (ATCC 12915) was determined resistant to tetracycline, chloramphenicol, and penicillin. The two strains of *Streptococcus* were either resistant (R) (*S. pneumoniae*) or intermediate resistant (IR) (*S. suis*) to tetracycline.

## 3. Discussion

Results in the current study indicated that butyric acid, valeric acid, and ProPhorce (the mixture of sodium formate and free formic acid, 40:60 *w*/*v*) had the strongest in vitro antimicrobial effects against *E. coli* and *Salmonella* strains, followed by the monoglycerides of SCFAs and lauric acid. However, sodium formate did not exhibit inhibitory effects on *E. coli* and *Salmonella* strains at the highest tested doses. Different trends were detected in the antimicrobial activities of the tested compounds against *Campylobacter* strains, as follows: butyric acid, valeric acid, and ProPhorce > sodium formate, monovalerin, monolaurin > monopropionin and monobutyrin. In addition, the strongest antimicrobial activities against G+ bacteria were observed in monolaurin, ProPhorce, butyric acid, and valeric acid. The weakest antimicrobial activities against G+ bacteria were observed in monopropionin and sodium formate, whereas monovalerin and monobutyrin were in the middle.

Short-chain fatty acids are fatty acids with a chain of less than six carbon atoms, which are primarily produced by hindgut fermentation of dietary fiber [22]. Propionic acid and butyric acid produced in the gastrointestinal tract of animals are considered particularly important metabolites that have antimicrobial effects on pathogenic bacteria [22,23]. The antimicrobial activities of butyric acid have been widely reported in previously published research to effectively inhibit G− and G+ bacteria, such as commensal *E. coli*, *Klebsiella pneumoniae*, *S.* Typhimurium, and *C. perfringens* [8,9,14,24,25]. It has been also reported that the valeric acid-producing bacteria *Oscillibacter valericigenes* were more abundant in the fecal samples of healthy people than people with Crohn’s disease [26], indicating valeric acid may also benefit intestinal health. Results in the current study suggest that valeric acid has similar antimicrobial activity against G− and G+ bacteria in comparison to butyric acid. The mode of action is likely due to the ability of these acids to penetrate bacterial cell membrane and to acidify cell cytoplasm, thus inhibiting bacterial growth [12,13,14]. Other mechanisms have been also proposed that organic acids could reduce ATP production by uncoupling electron transport, or they could interrupt nutrient uptake by disturbing bacterial cell membrane [11,27,28].

The present study also observed that ProPhorce exhibited stronger antimicrobial activities against G− and G+ bacteria compared with sodium formate. ProPhorce is a mixture of sodium formate and formic acid; therefore, current results indicate that formic acid likely has stronger in vitro antimicrobial activity than sodium formate. To that end, formic acid is the major component responsible for antimicrobial effects of ProPhorce in vitro. These results were not surprising because the antimicrobial activity of formic acid has been confirmed and widely reported against a broad range of bacterial strains, including *E. coli*, *S.* Typhimurium, *Campylobacter* strains, and *S. mutans* in previously published research [29,30,31,32]. Formic acid is a colorless liquid with pungent odor that has been commonly used in animal feed as an organic acidifier [6,33,34]. However, results from the current study suggest sodium formate has very limited antimicrobial activity in vitro.

Monoglycerides of SCFAs have several remarkable advantages compared with free SCFAs. They are more stable and have a less stringent odor compared with free SCFAs, increasing their potential as alternatives to antibiotics in animal feed. In addition, the ester forms of organic acids are digested and absorbed as lipids, which ensures they pass the low-pH stomach and successfully deliver their antimicrobial effects to the small intestine of animals. In the current study, monobutyrin and monovalerin exhibited comparable inhibitory effects on G− and G+ bacteria, although their antimicrobial activities were not as strong as their acid forms. However, monopropionin has weaker antimicrobial activities against G− and G+ bacteria compared with monobutyrin and monovalerin. Results of the present study were consistent with previously published research that indicated monobutyrin had antimicrobial effects on many *E. coli* strains, *S.* Typhimurium, and *C. perfringens* strains in vitro [25,35].

Medium chain fatty acids have recently attracted increased attention due to their potential antimicrobial activities and their potential ability to suppress the development of antibiotic-resistant genes in bacteria [21,36,37]. Lauric acid is a C12 fatty acid and has been indicated to have the strongest antimicrobial activity compared with other medium chain fatty acids [38,39,40]. Although Schlievert and Peterson [41] reported several G− bacteria, including *Salmonella* and *E. coli* strains, were not susceptible to monolaurin, results of the present study suggest monolaurin has similar or even stronger antimicrobial activity against *E. coli, S.* Typhimurium, and *C. jejuni* strains compared with monobutyrin. This could be due to different bacterial strains that have different susceptibility. These observations are consistent with a study reported by Anacarso et al. [35], in which 37 *E. coli* strains were highly susceptible to a blend containing monolaurin and monobutyrin, although the antimicrobial activity was not tested with individual monoglycerides in this study. In agreement with previously published research [35,41,42], the present study demonstrated that G+ bacteria were more susceptible to monolaurin than G− bacteria, with MIC values from 10 to 500 mg/L against G+ bacteria and MIC values from 600 to 10,000 mg/L against G− bacteria. It has also been reported that monolaurin actively inhibited the growth of *Staphylococcus, Streptococcus*, *Bacillus*, and several other G+ bacterial strains with relatively low MIC values [37,43]. As discussed above, the antimicrobial activities of fatty acids and their derivatives are mainly due to the disruption of bacterial cell membranes and the subsequent cell disorganization. However, the ability of medium chain fatty acids to disrupt cellular membranes has been demonstrated to vary among bacterial strains. This variation in susceptibility is likely due to the different outer membranes of the bacteria. For instance, G+ bacteria have cell walls composed of thick layers of peptidoglycan, whereas G− bacteria have a thin layer of peptidoglycan and an outer membrane that is primarily composed of lipopolysaccharides and proteins [40,44]. The O-side chains of lipopolysaccharides comprise an effective barrier for hydrophilic molecules, such as lipids [40,45]. In addition, these lipopolysaccharides are strongly connected, which makes it difficult for molecules to penetrate the outer membranes. This could be the reason that G− bacteria were less susceptible to monolaurin than G+ bacteria in the present study. The outer membrane of *Campylobacter* species expresses lipooligosaccharides that lack the O-side chain [46]; therefore, they are also more susceptible to monolaurin compared with *E. coli* and *S.* Typhimurium. Other mechanisms have been suggested for the antimicrobial effects of monolaurin on G+ bacteria, including the disturbance of toxin and exo-protein production at the transcriptional level or the regulation of bacterial signaling pathways that are critical for bacterial survival [37,41,47].

With the purpose of understanding antimicrobial susceptibility of bacterial strains in this study, the MIC values of antimicrobial drugs were also tested on the same strains of bacteria. All tested strains of *E. coli* and *Salmonella* exhibited multidrug resistance (i.e., resistant to ≥3 drugs). With the limited available MIC interpretive criteria, both strains of *Campylobacter* were determined resistant to ciprofloxacin, gentamicin, and tetracycline. The development of resistance to commonly used antibiotics by G− bacteria has gained increasing concern. For example, in 2012, the United States Department of Agriculture (USDA)’s national animal health monitoring system (NAHMS) isolated 1614 *E. coli* strains from swine production sites in 13 states that represented 91% of the U.S. pig inventory. Almost all *E. coli* isolated from swine (91.2%) were resistant to tetracycline (an antimicrobial drug used to treat pneumonia, certain skin infections, etc.), and more than one-third of the isolated *E. coli* were resistant to sulfisoxazole, a common sulfa antibiotic [48]. Interestingly, F18 *E. coli*, one of the most dominant types of pathogenic *E. coli* causing post-weaning diarrhea in piglets, was shown in this study to be susceptible in vitro to the organic acids and their derivatives, although the strain was determined resistant and intermediate resistant to multiple antimicrobial drugs. Post-weaning diarrhea accounts for 20–30% of cases of mortality in weanling pigs, causing huge economic loss in the pig industry [49,50]. Results of the present study suggest organic acid derivatives could be supplemented as antibiotic alternatives to prevent or control post-weaning diarrhea caused by F18 *E. coli* infection.

In regard to antimicrobial susceptibility of the tested G+ bacterial strains, broad resistance in *Enterococcus*, multidrug resistance in *C. perfringens*, and resistance to at least one drug in *Streptococcus* were observed in the present study. Interestingly, monolaurin at relatively low concentrations in our study inhibited the in vitro growth of these antimicrobial resistant pathogens. Taking *C. perfringens* as an example, this bacterial species is one of the most common foodborne pathogens in humans, and is also responsible for severe infections in animals, especially in poultry [33,51]. *C. perfringens*-induced necrotic enteritis may cause sudden death of broiler chickens, with mortality rates of up to 50% [52,53,54]. Subclinical *C. perfringens* infection also contributes to huge economic loss due to poor performance and high cost of medication and maintenance [55]. Although organic acids (i.e., formic acid, butyric acid, etc.) have been widely reported to control necrotic enteritis and to promote performance of chickens, the utilization of their derivatives are limited [33,56]. In summary, our results showed promising in vitro antimicrobial effects of tested organic acids and their derivatives against tested bacterial strains that are resistant to commonly used antimicrobial drugs. In vivo animal trials are needed to evaluate the efficacy of organic acid derivatives on animal health, such as in pigs and poultry.

## 4. Materials and Methods

### 4.1. Organic Acids and Their Derivatives

In vitro assays of antimicrobial activity against G− and G+ bacteria were performed on monopropionin, monobutyrin, monovalerin, monolaurin, butyric acid, valeric acid, sodium formate, and ProPhorce (a mixture of sodium formate and free formic acid with 40:60 *w/v*). All tested compounds were provided by Perstorp Waspik BV (Waspik, The Netherlands). Butyric acid, valeric acid, sodium formate, and ProPhorce are water-soluble and were directly mixed into culture broth at different tested concentrations based on previously published research [25,40] and industry recommendation. Monovalerin, monobutyrin, monopropionin, and monolaurin are not water-soluble. These compounds were first dissolved into ethanol prior to mixing into culture broth. The working concentrations (*v/v*) of ethanol in culture broth were optimized as 0.5% for monopropionin, 10% for monovalerin and monolaurin, and 20% for monobutyrin. The tested concentrations for individual organic acids and their derivatives are listed in Table 1.

### 4.2. Tested Bacterial Strains

Six G− bacterial strains and four G+ bacterial strains were used in these in vitro assays (Table 6). Among these bacterial strains, *E. coli* ATCC 25922 and *E. faecalis* ATCC 29212 are reference strains recommended by Clinical and Laboratory Standards Institute (CLSI) for antimicrobial susceptibility testing. These two strains and *C. jejuni* ATCC 33560 are also recommended as control strains for antimicrobial susceptibility testing by the European Committee on Antimicrobial Susceptibility Testing (EUCAST). *C. perfringens* ATCC 12915 is a control strain recommended by the British Society for Antimicrobial Chemotherapy (BSAC) for antimicrobial susceptibility testing. *S. pneumoniae* ATCC 49619 is a reference strain recommended by the EUCAST, the CLSI, and the BSAC. *S.* Typhimurium (ATCC 14028) has been used in control culture, media testing, preparatory test control, enteric research, emerging infectious disease research, pharmaceutical and personal care, and water testing according to the information from the American Type Culture Collection (ATCC).

*S. suis* ATCC 43765 is a strain isolated from pigs. The *E. coli* F18 strain is a pathogenic strain originally isolated from a field disease outbreak by the University of Illinois Veterinary Diagnostic Lab (isolate number: U.IL-VDL # 05-27242). *S.* Typhimurium (ID #4286) is a wild strain isolated from a cull dairy cow in California. *Campylobacter* (Campy 8DLIS D12-1) is a wild strain isolated from environmental water in California.

### 4.3. MIC Assays of G- Bacteria

The minimum inhibitory concentration (MIC) of individual organic acids against different bacterial strains was tested in triplicates using micro-broth dilution method [57,58,59]. For *E. coli* and *Salmonella* strains, four to five well-isolated fresh colonies were used to inoculate 2 mL brain heart infusion (BHI) broth and then incubated at 37 °C without CO_2_ for 2–6 h. The broth cultures were added dropwise to 0.85% NaCl to achieve a turbidity equivalent to a 0.5 McFarland nephelometer standard. Next, 10 µL of this bacterial solution was added to 40 µL cation-adjusted Mueller–Hinton broth and inoculated into 96-well plates containing serially diluted tested organic acids. Plates were then incubated at 37 °C without CO_2_ for 18–24 h. *Campylobacter* strains were retrieved by streaking on Trypticase soy agar with 5% sheep’s blood and incubating in a jar (Pack-Rectangular Jar, MGC, NY, USA) at 42 °C with Campy sachets (GasPak EZ Campy container System, BD, MD, USA). Fresh colonies were inoculated into BHI broth to prepare bacterial solutions and to perform the MIC assays in the same way as described above, except that the plates were incubated in a jar at 42 °C with Campy sachets for 18–24 h. *E. coli* ATCC 25922 was included in all assays testing G− bacteria. Culture broth without ethanol was used as medium control. Ethanol at designated concentrations equivalent to working concentrations in treated culture broth was used as solvent control. Medium control and solvent control were included for each bacterial strain in all assays. The MIC values were determined for individual organic acids and their derivatives as the minimum concentration that inhibits visible growth (e.g., turbidities, sediments) of bacteria. The MIC values were expressed as arithmetic means of triplicate tests and standard deviation (SD).

### 4.4. MIC Assays of G+ Bacteria

For *C. perfringens*, *S. suis*, and *S. pneumoniae* strains, bacterial solutions were prepared and MIC assays were performed using the same procedures as described above, except for the incubation conditions. Specifically, *C. perfringens* was incubated in a pouch (GasPak EZ Campy Gas Generating Pouch System, BD Diagnostics, Sparks, MD, USA) at 37 °C with anaerobic sachets (Anaero Pouch System, Mitsubishi Gas Chemical America, Inc. New York, NY, USA) for 18–24 h. *S. pneumoniae* was incubated in a pouch at 37 °C with Campy sachets for 18–24 h. *S. suis* was incubated at 37 °C without CO_2_ for 24 h. The *E. faecalis* ATCC 29212 was included in all assays testing G+ bacteria. Medium control and solvent control were included in all assays as well. The MIC values were determined for each organic acid as the minimum concentration that inhibits visible growth (e.g., turbidities, sediments) of bacteria.

### 4.5. Assay of Bacterial Susceptibility to Antimicrobial Drugs

Antimicrobial susceptibility of G− bacterial strains was tested against 24 antimicrobial drugs, and that of G+ bacterial strains was tested against 16 antimicrobial drugs, independently. These drugs represent current commonly used drugs in human and veterinary medicine based on the panel of antimicrobials of the National Antimicrobial Resistance Monitoring System (NARMS) tests (Table 2 and Table 4). Sensititre Gram-negative plate GN4F (G−) and Sensititre Gram-positive NARMS plate CMV3AGPF (G+) were purchased from Thermo Fisher Scientific (Waltham, MA, USA). The procedures for testing MIC of antimicrobial drugs followed the standard instructions provided in the plates and used same MIC methods as described above. Interpretation of the susceptibility of *E. coli*, *Salmonella*, *Enterococcus*, *Clostridium*, and *Streptococcus* was based on CLSI criteria [60]. Interpretation of the susceptibility of *Campylobacter* was based on the criteria for susceptibility testing used by Centers for Disease Control and Prevention (CDC) NARMS (https://www.cdc.gov/narms/antibiotics-tested.html).

## 5. Conclusions

Organic acids and their derivatives exhibited promising antimicrobial effects against both G− and G+ bacteria that were resistant to antimicrobial drugs in the current study. The order of overall antimicrobial strength, in descending order, was butyric acid, valeric acid, and formic acid > monovalerin, monolaurin, and monobutyrin > monopropionin and sodium formate. Specifically, SCFAs and formic acid were the most promising inhibitors of G− bacteria. G+ bacteria were highly susceptible to monolaurin at the concentration of 10 mg/L, and were also susceptible to SCFAs, formic acid, monobutyrin, and monovalerin. These compounds with promising in vitro antimicrobial activities may present a feasible alternative to antibiotic growth promoters in animal feed. The free forms of organic acids may exhibit high potential in the stomach, whereas the ester forms of organic acids may be able to deliver the benefits to the small intestine of animals. Additionally, it is speculated that bacteria do not develop resistance to organic acids as they have done to antimicrobial drugs. However, more research must be conducted to confirm this speculation. More in vivo research in livestock animals is necessary to test the efficacy of these in vitro effective organic acid derivatives as alternatives to antibiotics in feed.

## Figures and Tables

**Table 1:** Information of organic acids and their derivatives.

Compound	Form	Chemical Formula	Chemical Structure	Gravity, g/mL	Tested Concentration, mg/L
Butyric acid	Liquid	C_4_H_8_O_2_	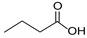	0.94	10, 250, 500, 1000, 2000, 2500, 3000, 3500
Valeric acid	Liquid	C_5_H_10_O_2_	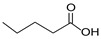	0.92	10, 250, 500, 1000, 2000, 2500, 3000, 3500
Sodium formate	Solid	NaHCOO	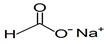	ND ^2^	500, 1000, 2000, 4000, 8000, 10,000, 12,500, 15,000, 17,500, 20,000, 25,000
ProPhorce ^1^	Liquid	NaHCOO CH_2_O_2_	-	1.36	10, 250, 500, 1000, 2000, 4000, 8000, 10,000
Monopropionin	Liquid	C_6_H_12_O_4_	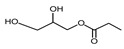	1.30	500, 1000, 2000, 2500, 3000, 3500, 5000, 7500, 10,000, 25,000
Monobutyrin	Liquid	C_7_H_14_O_4_	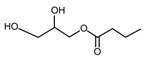	1.03	10, 250, 500, 1000, 2000, 2500, 3000, 3500, 5000, 10,000, 15,000, 20,000, 25,000, 30,000, 40,000, 50,000
Monovalerin	Liquid	C_8_H_16_O_4_	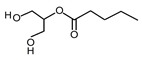	1.01	10, 250, 500, 1000, 2000, 2500, 3000, 3500, 5000, 10,000, 15,000, 20,000, 25,000
Monolaurin	Solid	C_15_H_30_O_4_	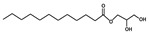	ND	10, 250, 500, 1000, 2000, 2500, 3000, 3500, 5000, 10,000, 15,000, 20,000, 25,000

^1^ ProPhorce is a mixture of sodium formate and free formic acid with 40:60 of *w*/*v* ratio. ^2^ ND: not detected due to the solid form.

**Table 2:** Minimum inhibitory concentrations (mg/L) (±SD) of organic acids and their derivatives on tested Gram-negative (G−) bacteria strains.

	*Escherichia coli*	*Escherichia coli*	*Salmonella enterica* Typhimurium	*Salmonella enterica* Typhimurium	*Campylobacter jejuni*	*Campylobacter jejuni*
Compound	ATCC 25922	F18	ATCC 14028	ID# 4286	ATCC 33560	Campy 8DLIS D12-1
Butyric acid	2300 (±250)	2500 (±0)	2500 (±0)	2300 (±250)	800 (±300)	500 (±0)
Valeric acid	2700 (±400)	2800 (±400)	2700 (±300)	2600 (±200)	500 (±0)	700 (±300)
Sodium formate	>20,000	>20,000	>20,000	>20,000	2000 (±0)	2000 (±0)
ProPhorce ^1^	2000 (±0)	2200 (±700)	2200 (±700)	2200 (±700)	700 (±300)	1000 (±0)
Monopropionin	>10,000	>10,000	>10,000	≥10,000	≥10,000	>10,000
Monobutyrin	15,000 (±0)	10,000 (±0)	11,700 (±2400)	10,000 (±0)	>50,000 ^a^	10,000 (±0)
Monovalerin	6700 (±2400)	5000 (±0)	10,000 (±5000)	15,000 (±0)	2500 (±1300)	3700 (±900)
Monolaurin	10,000 (±0)	10,000 (±0)	10,000 (±0)	10,000 (±0)	600 (±100)	5000 (±0)

^1^ ProPhorce is a mixture of sodium formate and free formic acid with 40:60 of *w*/*v* ratio; ^a^ no higher concentration was tested due to the elevated concentrations of ethanol.

**Table 3:** Antimicrobial susceptibility of tested G- bacteria strains.

Antimicrobial Drug	Range of Concentrations (mg/L)	*Escherichia coli*	*Escherichia coli*	*Salmonella enterica* Typhimurium	*Salmonella enterica* Typhimurium	*Campylobacter jejuni*	*Campylobacter jejuni*
		ATCC 25922	F18	ATCC 14028	ID# 4286	ATCC 33560	Campy 8DLIS D12-1
Amikacin	8–32	ORC	S	ORC	ORC	NA	NA
Piperacillin/tazobactam constant 4	8/4–128/4	R	S	R	R	NA	NA
Tigecycline	1–8	NA	NA	NA	NA	NA	NA
Ticarcillin/clavulanic acid constant 2	8/2–64/2	IR	S	ORC	IR	NA	NA
Levofloxacin	1–8	R	R	R	R	NA	NA
Nitrofurantoin	32–64	ORC	S	ORC	ORC	NA	NA
Tetracycline	4–8	ORC	ORC	ORC	ORC	R	R
Doripenem	0.5–4	R	S	R	R	NA	NA
Minocycline	1–8	ORC	IR	ORC	ORC	NA	NA
Ertapenem	0.25–8	R	IR	R	R	NA	NA
Trimethoprim/sulfamethoxazole	2/38–4/76	R	R	R	R	NA	NA
Imipenem	0.5–8	R	IR	R	R	NA	NA
Piperacillin	16–64	ORC	IR	ORC	ORC	NA	NA
Meropenem	0.5–8	R	S	R	R	NA	NA
Gentamicin	2–8	ORC	S	ORC	ORC	R	R
Cefazolin	1–16	R	R	R	R	NA	NA
Tobramycin	2–8	ORC	S	ORC	ORC	NA	NA
Ceftazidime	1–16	R	R	R	R	NA	NA
Ampicillin/sulbactam 2:1 ratio	4/2–16/8	ORC	IR	ORC	IR	NA	NA
Aztreonam	1–16	NA	NA	NA	NA	NA	NA
Ampicillin	8–16	ORC	ORC	ORC	IR	NA	NA
Cefepime	4–32	R	SSD	R	R	NA	NA
Ciprofloxacin	0.5–2	ORC	S	R	R	R	R
Ceftriaxone	0.5–32	R	R	R	R	NA	NA

S: susceptible; SSD: susceptible-dose dependent; IR: intermediate resistant; R: resistant; ORC: out range of concentration; NA: no interpretative criteria for this bacterium/antimicrobial combination currently available.

**Table 4:** Minimum inhibitory concentrations (mg/L) (±SD) of organic acids and their derivatives on tested Gram-positive (G+) bacteria strains.

	*Enterococcus faecalis*	*Clostridium perfringens*	*Streptococcus pneumoniae*	*Streptococcus suis*
Compound	ATCC 29212	ATCC 12915	ATCC 49619	ATCC 43765
Butyric acid	2000 (±0)	1200 (±400)	1000 (±0)	700 (±2400)
Valeric acid	2000 (±0)	1300 (±700)	1000 (±0)	1000 (±0)
Sodium formate	>20,000	18,800 (±7100)	15,800 (±24,00)	11,000 (±7100)
ProPhorce ^1^	1000 (±0)	1000 (±0)	1000 (±0)	1900 (±3400)
Monopropionin	>10,000	11,300 (±6400)	>25,000	>25,000
Monobutyrin	10,000 (±0)	2600 (±1300)	7700 (±2900)	7800 (±2500)
Monovalerin	10,000 (±0)	3100 (±1200)	2400 (±400)	2000 (±700)
Monolaurin	500 (±0)	300 (±400)	10 (±0)	400 (±800)

^1^ ProPhorce is a mixture of sodium formate and free formic acid with 40:60 of *w*/*v* ratio.

**Table 5:** Antimicrobial susceptibility of tested G+ bacteria strains.

Antimicrobial Drugs	Range of Concentrations (mg/L)	*Enterococcus faecalis*	*Clostridium perfringens*	*Streptococcus pneumoniae*	*Streptococcus suis*
		ATCC 29212	ATCC 12915	ATCC 49619	ATCC 43765
Tigecycline	0.015–0.5	NA	NA	NA	NA
Erythromycin	0.25–8	IR	NA	S	S
Tetracycline	1–32	R	R	R	IR
Ciprofloxacin	0.12–4	R	NA	NA	NA
Chloramphenicol	2–32	R	R	S	S
Penicillin	0.25–16	R	R	NA	ORC
Daptomycin	0.25–16	NA	NA	S	S
Vancomycin	0.25–32	R	NA	S	S
Streptomycin	512–2048	R	NA	NA	NA
Nitrofurantoin	2–64	ORC	NA	NA	NA
Tylosin tartrate	0.25–32	R	NA	NA	NA
Gentamicin	128–1024	R	NA	NA	NA
Quinupristin/dalfopristin	0.5–32	R	NA	S	S
Lincomycin	1–8	S	NA	NA	NA
Linezolid	0.5–8	R	NA	S	S
Kanamycin	128–1024	R	NA	NA	NA

S: susceptible; IR: intermediate resistant; R: resistant; ORC: out range of concentration; NA: no interpretative criteria for this bacterium/antimicrobial combination currently available.

**Table 6:** Information of tested bacterial strains.

Species	Strain Designation	Gram Stain	Strain Type
*Escherichia coli*	ATCC 25922	G−	reference
*Escherichia coli*	F18	G−	wild
*Salmonella enterica* Typhimurium	ATCC 14028	G−	reference
*Salmonella enterica* Typhimurium	Sample ID #4286	G−	wild
*Campylobacter jejuni*	ATCC 33560 (CIP 702)	G−	reference
*Campylobacter jejuni*	Campy 8DLIS D12-1	G−	wild
*Enterococcus faecalis*	ATCC 29212	G+	reference
*Clostridium perfringens*	ATCC 12915	G+	reference
*Streptococcus pneumoniae*	ATCC 49619	G+	reference
*Streptococcus suis*	ATCC 43765	G+	wild
