# Correction: Kovanda, L., et al. In Vitro Antimicrobial Activities of Organic Acids and Their Derivatives on Several Species of Gram-Negative and Gram-Positive Bacteria. *Molecules* 2019, *24*, 3770

**DOI:** 10.3390/molecules25122787

**Published:** 2020-06-17

**Authors:** Lauren Kovanda, Wen Zhang, Xiaohong Wei, Jia Luo, Xixi Wu, Edward Robert Atwill, Stefan Vaessen, Xunde Li, Yanhong Liu

**Affiliations:** 1 Department of Animal Science, University of California, Davis, CA 95616, USA; llkovanda@ucdavis.edu; 2 School of Life Science, Ningxia University, Yinchuan 750021, China; nx_zhangwen@163.com (W.Z.); sophia920209@163.com (J.L.); wuxixivip@163.com (X.W.); 3 School of Veterinary Medicine, University of California, Davis, CA 95616, USA; xhcwei@ucdavis.edu (X.W.); ratwill@ucdavis.edu (E.R.A.); 4 Perstorp Waspik BV, 5165 NH Waspik, The Netherlands; Stefan.Vaessen@perstorp.com

The authors wish to make the following corrections to this paper published in *Molecules* [1]. 

Monopropionin, monobutyrin, and monovalerin described in the original article consist of approximately 50% monoglycerides, 35% diglycerides, 5% triglycerides, and 10% glycerol, respectively. To increase the accuracy of the product description, monopropionin, monobutyrun, and monovalerin were changed to propionate glycerides, butyrate glycerides, and valerate glycerides throughout the article. Monoglycerides were changed to glyceride esters in the first and fourth paragraph of the discussion section. The chemical structures in Table 1 only referred to monoglycerides.

The changes have no impact on the conclusions of the paper. We apologize for any inconvenience to our readers.
